# Cancer Incidence Among Swedish Seafarers Between 1985 and 2020

**DOI:** 10.1002/ajim.70126

**Published:** 2026-08-15

**Authors:** Maria Wallin, Helena P. Eriksson, Karl Forsell, Eva Andersson

**Affiliations:** ^1^ Department of Occupational and Environmental Medicine Sahlgrenska Academy University of Gothenburg Gothenburg Sweden; ^2^ Department of Occupational and Environmental Medicine Sahlgrenska University Hospital Gothenburg Region Västra Götaland Sweden

**Keywords:** cancer incidence, cohort, epidemiology, occupation, register study, seafarer, Sweden

## Abstract

**Background:**

Several studies from different countries have shown that merchant seafarers have an increased cancer risk compared to the general population. The aim of this study was to provide updated information on cancer incidence in a cohort of Swedish seafarers.

**Methods:**

A cohort consisting of all Swedish seafarers from the Swedish Seafarers' Register 1985–2013 was linked to the Swedish Cancer Register 1985–2020. Seafarers with at least 5 years of sea service (*N* = 38,030; 76% men and 24% women) were included in the analyses. Standardized incidence ratios (SIR) were calculated for cancer by specific site, period of first signing, job category, type of vessel, and gender, using cancer incidence in the general population as reference.

**Results:**

In seafarers who had worked on different types of vessels (not solely passenger ferries), there were 5076 cases of cancer during the follow‐up period, and total cancer risk was increased (SIR 1.14; 95% confidence interval [CI] 1.11–1.17). Most cases occurred in seafarers who had their first signing before 1985. The risk of lung cancer was increased in all job categories, especially in engine room crew members (SIR 1.98; 95% CI 1.53–2.57). There were 16 mesothelioma cases in engine officers (SIR 3.75; 95% CI 2.30–6.12).

**Conclusions:**

This cohort study of seafarers in Sweden confirms that Swedish seafarers had an increased overall cancer risk compared with the general population, particularly those signing before 1985 and working on different types of vessels.

## 1 Introduction

Several studies from northern Europe have shown that merchant seafarers have an increased cancer risk compared to the general population [1, 2, 3, 4, 5, 6, 7, 8]. To some extent, the increased cancer risk is probably derived from seafarers' lifestyle factors such as tobacco smoking and alcohol consumption, as previous studies from the Nordic countries have indicated that seafarers used to smoke more and drink more alcohol than the rest of the working population [1]. In recent years, however, the smoking habits of Swedish seafarers seem to have become more similar to those of the general population [9]. For alcohol consumption habits, there was no legislation restricting boating under the influence of alcohol in Sweden before 1967, and it was not until the 1990s that agreements were concluded between associations for ship owners and seafarers on alcohol and drugs [10].

However, part of the increased risk is likely due to occupational exposure to carcinogenic factors. For example, the incidence of lung cancer and mesothelioma is higher in engine crew [2]. Asbestos is a well‐known occupational carcinogen that has previously been widely used in many merchant vessels, for example in the engine room. Exposure to asbestos is considered to be the cause of almost all cases of mesothelioma, and it is also associated with an increased risk of lung cancer, especially in smokers [11]. Other potential carcinogens present in seafarers' working environment include polyaromatic hydrocarbons in diesel engine exhaust fumes, soot, and oils, which may increase the risk of lung cancer, skin cancer, and bladder cancer [7]. This type of exposure is particularly common among those who work in the engine room [9]. Deck crew workers on tankers may be exposed to benzene, which has been linked to various types of blood cancer [9, 12, 13]. Tankers may carry benzene in the cargo either as the main product or as part of a petroleum product [12]. In a previous study of the work environment in the Swedish merchant fleet, 10% of the seafarers reported that they did not have access to necessary protective equipment [9]. In addition, many seafarers work frequently outdoors and are thus exposed to UV radiation from the sun, which increases the risk of skin cancer [14]. It is also very common for seafarers to work on the night‐shift, which is a risk factor for breast cancer (IARC group 2A) [15, 16].

In our previous study on cancer incidence in Swedish seafarers between 1985 and 2011, total cancer risk was increased in men, but not in women [7]. Lung cancer was increased in both male and female seafarers. There was also a tendency towards decreasing cancer incidence at the end of this period.

The aim of the current study was to update the assessment of cancer risk in Swedish male and female seafarers using the same cohort, but with a longer follow‐up, and only including those who had worked as seafarers for at least five years. The cohort was divided into two main groups; those who first signed before 1985, and those who first signed in 1985 or later. In addition, the seafarers were divided into subgroups based on their job category, and whether they had worked on different types of vessels or solely on passenger ferries.

## 2 Materials and Methods

### 2.1 Study Design and Population

This is a longitudinal cohort study on seafarers included in the Swedish Seafarers' Register, which is administered by the Swedish Transport Agency. In Sweden, it is mandatory to register all seafarers who work on Swedish merchant vessels with a gross tonnage of 20 tons or more. The Seafarers' Register contains detailed information on every seafarer's signing, such as the dates, type of vessel, job category (deck, engine, service, other), and position on board (officer, crew/rating). The register was digitalized in 1985, and seafarer data has been transferred from the old register to the new digital register for those still working as seafarers in 1985. We decided to divide the cohort into two groups, those who started working as seafarers before 1985 and those who started 1985 or later, as these two groups differ in several respects. The most important reason is that we do not have complete follow‐up of all seafarers who started working before 1985, because only those who were still working as seafarers in 1985 were included in the digital register. This implies that those who stopped working as seafarers before 1985, for example due to illness or switching to a land‐based job, are not included in the cohort. However, in the group that started working in 1985 or later, all seafarers are included in the digital register and therefore in the cohort. In addition, for seafarers who solely worked on passenger ferries, the data in the Seafarers' Register is not complete for the period before 1985, and we therefore only report cancer incidence ratios in this group for those with registered signings that started 1985 or later.

The cohort initially consisted of all 105,098 seafarers in the register [7, 17]. We excluded seafarers who died before 1985 (*n* = 101), foreign nationals without a Swedish personal identity number (*n* = 9536), and those with re‐used or incorrect personal identity numbers (*n* = 51). Seafarers who had their first signing after 2013 (*n* = 1870), those with less than 30 days of sea service (*n* = 7916), seafarers born before 1920 (*n* = 198), and those who had their last signing before 1980 (*n* = 257) were also excluded. Finally, to achieve a longer exposure time, we decided to exclude those with less than five years of sea service in the Seafarers' register (*n* = 47,139). This automatically resulted in a lag time of at least five years from first exposure to diagnosis.

The data from the Seafarers' Register was linked to the Swedish Cancer Register by the National Board of Health and Welfare using the seafarers' personal identity number and then anonymized before we were given access to data. Cancer diagnoses were given according to the 7th International Classification of Diseases (ICD‐7).

We divided seafarers into two categories: those who had worked *solely on passenger ferries* and those who had worked on *different types of vessels* (Supporting Information: Table I). Approximately half of the seafarers had worked exclusively on passenger ferries, and the other half had worked on several types of vessels, and the working conditions are likely quite different for these two groups. For example, those who work on passenger ferries probably have more regular work, and they are at sea for shorter periods. The seafarers were first classified into the following job categories: *solely service, ever officer*, or *crew*. The category *ever officer* was further divided into two subcategories: *ever engine officer* (if ever having worked as engine officer) or *deck officer*. Crew members were correspondingly classified as *engine crew* (if ever having worked in the engine room but not as an officer), or *deck crew* (if having worked as deck crew but not as an officer). Seafarers in the category *solely service* had worked exclusively in the service section of a vessel (Supporting Information: Tables I–II).

### 2.2 Statistical Analysis

Person‐years at risk were calculated from five years after first sea service, or from 1985 if earlier, until first emigration, time of death, age of 85 years, or at the end of follow‐up on December 31, 2020. The person‐years were stratified by gender, five‐year age groups, and one‐year calendar periods. The expected numbers of cancer cases for these strata were calculated using the Swedish general population as reference. Standardized Incidence Ratios (SIRs) were calculated by dividing the number of observed cases for each cancer diagnosis with the expected number, and 95% confidence intervals (CI) were calculated assuming a Poisson distribution for the number of observed cancer cases if more than three cases. For seafarers working on different types of vessels, SIRs were analyzed stratifying for gender and start of sea service (before 1985, or 1985 and later, i.e., before follow‐up started or during follow‐up).

Further analyses stratified by job category (both genders combined) among those starting before 1985: deck officer, ever engine officer, deck crew, engine crew, and solely service. For seafarers working solely on passenger ferries, we only analyzed those starting 1985 or later by job category (both genders combined). Seafarers who had signed for the first time in 1985 or later (both those who had worked on different types of vessels and those who had worked solely on passenger ferries) were classified into one of three job categories in the main tables due to the low number of cases in these groups: solely service, ever officers, or crew. However, for a few specific sites of cancer (mouth‐pharynx, lung, prostate, and urinary bladder), we did additional analyses, classifying also the seafarers who had signed for the first time in 1985 or later into five job categories, as shown in the Supplement Table III. Calculations were made using STATA SE (release 17, Stata Statistical Software, College Station, TX, USA), and SAS (version 9.4). The Regional Ethical Committee of Gothenburg approved the study (Dnr 193‐13) and the Swedish Ethical Review Authority approved the update of the study (Dnr 2023‐06423‐02). Informed consent was not relevant as this was a register study on anonymized data.

## 3 Results

### 3.1 Study Population

The general characteristics of the cohort are shown in Table 1. The cohort consisted of 38,030 seafarers, 24% women and 76% men. Deck officers or deck crew made up the largest group of the different job categories among the seafarers, 42%, and 24% were classified as engine officers or engine crew, and 34% in catering or service.

**Table 1:** Characteristics of the cohort of Swedish seafarers with at least five years of sea service (with registered signings) before 2015, total and by gender, with follow up between 1985 and 2020. Column percentages based on total numbers are given.

	Total	Men	Women
	**Person‐years**	**Person‐years**	**Person‐years**
Person‐years of follow‐up for seafarers after 5 years of service	908,454	701,637	206,817
Start before 1985	576,779	483,997	92,782
Start during 1985–2013	331,675	217,640	114,035
	***N* (%)**	***N* (%)**	***N* (%)**
**Total numbers of seafarers**	**38,030**	**28,995**	**9035**
Deck, total	16,038 (42%)	14,660 (51%)	1378 (15%)
Deck officers, not engine	8570 (23%)	8194 (28%)	376 (4%)
Deck crew, not engine	7468 (20%)	6466 (22%)	1002 (11%)
Engine, total	9122 (24%)	8636 (31%)	186 (2%)
Engine officers, ever	5786 (15%)	5713 (20%)	73 (0.8%)
Engine crew	3336 (9%)	3223 (11%)	113 (1.3%)
Solely catering/service	12,870 (34%)	5399 (19%)	7471 (83%)
Vital status Dec 31, 2020			
Alive	28,638 (75%)	21,022 (73%)	7616 (84%)
Dead	6211 (16%)	5475 (19%)	736 (8%)
Emigrated	3181 (8%)	2498 (9%)	683 (8%)
Age at start of follow‐up			
< 30 years	15,680 (41%)	11,128 (38%)	4552 (50%)
30−59 years	21,186 (56%)	16,929 (58%)	4257 (47%)
≥ 60 years	1164 (3%)	938 (3%)	226 (3%)
Time from first to last sea service until 2015			
5−10 years	11,121 (29%)	6822 (24%)	4299 (48%)
10−20 years	10,774 (28%)	7839 (27%)	2935 (32%)
> 20 years	16,135 (42%)	14,334 (49%)	1801 (20%)
Age at end of follow‐up			
< 30 years	723 (2%)	517 (2%)	206 (2%)
30−59 years	18,336 (48%)	13,158 (45%)	5178 (57%)
≥ 60 years	18,971 (50%)	15,320 (53%)	3651 (40%)
Seafarers with start before 1985, age at end of follow up			
< 30 years	137 (0.4%)	112 (0.4%)	25 (0.3%)
30−59 years	4692 (12%)	4031 (14%)	661 (7%)
≥ 60 years	15,187 (40%)	12,776 (44%)	2411 (27%)
Seafarers with start 1985 or later, age at end of follow up			
< 30 years	586 (1.5%)	405 (1.4%)	181 (2%)
30−59 years	13,644 (36%)	9127 (31%)	4517 (50%)
≥ 60 years	3784 (10%)	2544 (9%)	1240 (14%)

### 3.2 Overall Results

In seafarers who had worked on different types of vessels (i.e., not solely on passenger ferries), 5076 cases of cancer occurred during the follow‐up period, 4647 cases in men and 429 in women (Table 2). Total cancer risk was increased (SIR 1.14; 95% CI 1.11–1.17). Most cases (91%) occurred in seafarers who had their first registered signing before 1985. After stratification for gender and period of first signing, the total cancer risk was only significant in men signing before 1985 (SIR 1.16, 95% CI 1.12–1.19). There was an increased risk of cancer in the mouth, pharynx, esophagus, stomach, liver, larynx, lung, pleura, and urinary bladder (all seafarers, genders combined, who had worked on different vessels; Table 2). The incidence of lung cancer was significantly increased among women signing in 1985 or later (SIR 3.47; 95% CI 1.97–6.10), as were the risks for lip and thyroid cancer among men (SIR 4.88; 95% CI 1.83–13.0, and SIR 2.10; 95% CI 1.09–4.03, respectively, few cases; Table 2). For stomach cancer, there was a significantly increased risk for both women and men signing before 1985, and for men signing 1985 or later (Table 2).

**Table 2:** Standardized incidence ratios (SIR) 1985–2020 by specific site for cancer (ICD‐7), year of first signing (before 1985 or 1985–2013) and gender among Swedish seafarers with five years or more of registered signings and working on different types of vessels (not solely passenger ferries).

	Numbers (person‐years)	Signing before 1985, men *N* = 15,693 (447,406 yrs)			Signing 1985‐2013, men *N* = 7589 (136,702 yrs)			Signing before 1985, women *N* = 1699 (50,636 yrs)				Signing 1985‐2013, women *N* = 1255 (23,039 yrs)			All seafarers, both genders N = 26,236 (657,783 yrs)			
ICD‐7	**Cancer site**	O	**SIR**	95% CI	O	**SIR**	95% CI	O	**SIR**	95% CI		O	**SIR**	95% CI	O	SIR	95% CI	
140‐209	All sites	4246	**1.16**	**1.12–1.19**	401	**1.08**	0.98–1.19	352	**1.05**	0.95–1.17		77	**1.00**	0.80–1.25	5076	**1.14**	**1.11–1.17**	
140	Lip	17	**1.37**	0.85–2.21	4	**4.88**	**1.83–13.0**	0		exp 0.66		0		exp 0.09	21	**1.51**	0.98–2.31	
141‐144	Mouth	60	**1.35**	**1.05–1.74**	9	**1.64**	0.85–3.15	4	**1.33**	0.50–3.53		0		exp 0.69	73	**1.36**	**1.08–1.71**	
145‐148	Pharynx	65	**1.92**	**1.51–2.45**	4	**0.90**	0.34–2.41	3		exp 1.29		1		exp 0.31	73	**1.83**	**1.46–2.31**	
150	Esophagus	78	**1.73**	**1.38–2.15**	5	**1.34**	0.56–3.22	4	**2.88**	**1.08–7.69**		1		exp 0.19	88	**1.74**	**1.41–2.15**	
151	Stomach	95	**1.23**	**1.00–1.50**	11	**1.82**	**1.01–3.29**	11	**2.54**	**1.41–4.58**		1		exp 0.65	118	**1.33**	**1.11–1.60**	
152	Small intestine	24	**1.06**	0.71–1.59	2		exp 2.52	0		exp 1.69		0		exp 0.32	26	**0.96**	0.65–1.41	
153	Colon	259	**1.04**	0.92–1.18	26	**1.14**	0.78–1.68	24	**1.07**	0.72–1.60		1		exp 3.37	310	**1.04**	0.93–1.16	
154	Rectum	177	**1.08**	0.94–1.26	20	**1.31**	0.84–2.02	11	**0.97**	0.54–1.76		3		exp 1.90	211	**1.10**	0.97–1.26	
155.0	Liver	66	**1.36**	**1.07–1.74**	5	**0.96**	0.40–2.30	4	**1.83**	0.69–4.87		2		exp 0.36	77	**1.37**	**1.10–1.71**	
157	Pancreas	79	**1.00**	0.80–1.24	8	**1.21**	0.60–2.41	6	**0.87**	0.39–1.93		0		exp 0.96	93	**0.99**	0.81–1.21	
160	Nasal cavity	5	**0.89**	0.37–2.14	0		exp 0.71	2		exp 0.36		0		exp 0.08	7	**1.04**	0.49–2.17	
161	Larynx	47	**1.92**	**1.44–2.56**	1		exp 1.82	2		exp 0.45		0		exp 0.08	50	**1.86**	**1.41–2.46**	
162.1	Lung	447	**1.58**	**1.44–1.73**	18	**0.91**	0.57–1.44	45	**1.88**	**1.40‐2.51**		12	**3.47**	**1.97–6.10**	522	**1.58**	**1.45–1.72**	
162.2	Pleura (mesothelioma)	27	**1.80**	**1.23–2.62**	1		exp 0.90	0		exp 0.26		0		exp 0.03	28	**1.73**	**1.19–2.50**	
170	Breast	5	**0.87**	0.36–2.09	1		exp 0.72	95	**0.84**	0.69–1.03		23	**0.76**	0.50–1.14	124	**0.83**	**0.70–0.99**	
171	Cervix uteri							6	**0.85**	0.38–1.88		3		exp 3.31	9	**0.87**	0.45–1.66	
172	Uterus							14	**0.77**	0.46–1.30		1		exp 2.69	15	**0.72**	0.43–1.19	
175	Ovaries							15	**1.18**	0.71–1.96		5	**2.14**	0.89–5.15	20	**1.33**	0.86–2.07	
177	Prostate	1176	**1.01**	0.96–1.07	88	**0.88**	0.72–1.09								1264	**1.00**	0.95–1.06	
178	Testis	20	**0.87**	0.56–1.36	20	**1.13**	0.73–1.75								40	**0.99**	0.72–1.34	
180	Kidney	102	**0.89**	0.74–1.09	19	**1.49**	0.95–2.34	6	**0.94**	0.42–2.10		2		exp 1.05	129	**0.96**	0.81–1.14	
181	Urinary bladder	302	**1.19**	**1.06–1.33**	23	**1.17**	0.78–1.76	11	**1.43**	0.79–2.59		1		exp 1.04	337	**1.20**	**1.07–1.33**	
190	Melanoma	217	**1.12**	0.98–1.28	36	**1.14**	0.82–1.58	14	**0.79**	0.47–1.34		7	**1.04**	0.50–2.19	274	**1.10**	0.98–1.24	
191	Other skin	336	**1.12**	**1.01–1.25**	19	**0.84**	0.53–1.31	20	**1.18**	0.76–1.83		1		exp 2.65	376	**1.10**	0.99–1.22	
193	Brain and nervous system	97	**1.05**	0.86–1.28	17	**1.02**	0.63–1.64	9	**0.82**	0.43–1.57		3		exp 3.26	126	**1.02**	0.86–1.21	
194	Thyroid	16	**0.98**	0.60–1.60	9	**2.10**	**1.09–4.03**	4	**0.98**	0.37–2.62		0		exp 2.31	29	**1.08**	0.75–1.55	
195	Other endocrine glands	39	**0.86**	0.63–1.18	9	**1.06**	0.55–2.03	8	**0.98**	0.49–1.97		3		exp 2.58	59	**0.91**	0.71–1.18	
197	Connective tissue	24	**1.10**	0.74–1.64	1		exp 3.25	0		exp 1.78		0		exp 0.45	25	**0.91**	0.62–1.35	
199	Other and unspecified site	104	**1.31**	**1.08–1.59**	9	**1.52**	0.79–2.92	5	**0.56**	0.23–1.34		1		exp 0.89	119	**1.25**	**1.04–1.49**	
200, 202	Non‐Hodgkin's lymphoma	128	**0.96**	0.81–1.14	10	**0.65**	0.35–1.20	7	**0.76**	0.36–1.60		2		exp 1.73	147	**0.92**	0.78–1.08	
201	Hodgkin's disease	7	**0.60**	0.29–1.27	5	**1.39**	0.58–3.35	0		exp 0.80		0		exp 0.46	12	**0.73**	0.41–1.29	
203	Multiple myeloma	38	**0.74**	0.54–1.02	2		exp 4.69	4	**1.16**	0.43–3.08		1		exp 0.52	45	**0.75**	0.56–1.01	
204‐209	Leukemia	124	**0.93**	0.78–1.11	14	**0.91**	0.54–1.53	4	**0.41**	0.15–1.10		3		exp 1.94	145	**0.91**	0.77–1.07	

*Note:* Observed cases (O) after 5 years latency from start of sea service and 95% confidence intervals (CI) are given. Expected number of cases is shown if three cases or less.

### 3.3 Job Category

Among those who had worked on different types of vessels, lung cancer was significantly increased in all job categories (Table 3). Mesothelioma was significantly increased only in those who had ever worked as engine officers (SIR 3.75; 95% CI 2.30–6.12; Table 3). The crew and service personnel, but not the officers, had an increased risk of liver and larynx cancer, and the engine crew had a significantly increased risk of stomach cancer (SIR 1.80, 95% CI 1.07–3.04; Table 3). Some cancer forms, such as cancer of the lip, urinary bladder, and prostate, as well as melanoma and other types of skin cancer, were only significantly increased in deck officers (Table 3).

**Table 3:** Standardized incidence ratios (SIR) 1985–2020 by specific site for cancer (ICD‐7) and job category among Swedish seafarers (genders combined) with 5 years or more of registered signings, with start of sea service before 1985, and working on different types of vessels (not solely passenger ferries).

	Numbers (person‐years)	Ever engine officer *N* = 4019 (115,528 yrs)			Deck officer *N* = 5383 (153,955 yrs)			Deck crew* *N* = 3469 (100,208 yrs)			Engine crew *N* = 1827 (52,066 yrs)			Solely service N = 2694 (76,285 yrs)			All categories *N* = 17 392 (498,042 yrs)		
ICD‐7	**Cancer site**	O	**SIR**	95% CI	O	**SIR**	95% CI	O	**SIR**	95% CI	O	**SIR**	95% CI	O	**SIR**	95% CI	O	**SIR**	95% CI
140‐209	All sites	1180	**1.16**	**1.09–1.23**	1682	**1.25**	**1.19–1.31**	649	**0.98**	0.91–1.06	427	**1.13**	**1.03–1.24**	660	**1.11**	**1.03–1.20**	4598	**1.15**	**1.12–1.18**
140	Lip	2		exp 3.47	9	**1.95**	**1.01–3.74**	3		exp 2.04	3		exp 1.23	0		exp 1.66	17	**1.31**	0.81–2.10
141‐144	Mouth	12	**0.98**	0.56–1.72	21	**1.33**	0.87–2.04	12	**1.45**	0.82–2.54	8	**1.69**	0.84–3.37	11	**1.74**	0.96–3.14	64	**1.35**	**1.06–1.72**
145‐148	Pharynx	18	**1.96**	**1.23–3.11**	26	**2.22**	**1.51–3.26**	13	**2.00**	**1.16–3.44**	6	**1.63**	0.73–3.62	5	**1.26**	0.52–3.02	68	**1.94**	**1.53–2.46**
150	Esophagus	24	**1.89**	**1.27–2.82**	26	**1.59**	**1.08–2.34**	15	**1.95**	**1.17–3.23**	8	**1.73**	0.86–3.45	9	**1.73**	0.90–3.33	82	**1.76**	**1.42–2.19**
151	Stomach	30	**1.39**	0.97–1.98	29	**1.01**	0.70–1.45	19	**1.45**	0.93–2.28	14	**1.80**	**1.07–3.04**	14	**1.33**	0.79–2.24	106	**1.30**	**1.07–1.57**
152	Small intestine	5	**0.80**	0.33–1.91	6	**0.74**	0.33–1.64	10	**2.43**	**1.31–4.52**	2		exp 0.85	1		exp 0.30	24	**0.99**	0.66–1.48
153	Colon	76	**1.09**	0.87–1.37	106	**1.15**	0.95–1.39	34	**0.78**	0.56–1.10	25	**0.99**	0.67–1.46	42	**1.03**	0.76–1.40	283	**1.04**	0.93–1.17
154	Rectum	54	**1.19**	0.91–1.56	60	**1.01**	0.78–1.30	24	**0.84**	0.56–1.26	24	**1.44**	0.97–2.15	26	**1.10**	0.75–1.61	188	**1.08**	0.94–1.25
155.0	Liver	14	**1.04**	0.62–1.76	11	**0.63**	0.35–1.15	23	**2.63**	**1.75–3.96**	10	**1.99**	**1.07–3.70**	12	**1.99**	**1.13–3.50**	70	**1.38**	**1.10–1.75**
157	Pancreas	20	**0.90**	0.58–1.39	27	**0.93**	0.64–1.35	12	**0.86**	0.49–1.52	4	**0.49**	0.18–1.30	22	**1.72**	**1.13–2.61**	85	**0.99**	0.80–1.22
160	Nasal cavity	2		exp 1.55	3		exp 2.04	0		exp 1.01	0		exp 0.59	2		exp 0.79	7	**1.17**	0.56–2.46
161	Larynx	10	**1.46**	0.78–2.70	12	**1.36**	0.77–2.39	12	**2.92**	**1.66–5.14**	6	**2.39**	**1.07–5.32**	9	**3.48**	**1.81–6.69**	49	**1.97**	**1.49–2.60**
162.1	Lung	120	**1.50**	**1.25–1.79**	158	**1.50**	**1.28–1.75**	76	**1.58**	**1.26–1.98**	57	**1.98**	**1.53–2.57**	81	**1.80**	**1.45–2.24**	492	**1.60**	**1.47–1.75**
162.2	Pleura (mesothelioma)	16	**3.75**	**2.30–6.12**	3	**0.55**	0.18–1.70	3	**1.23**	0.40‐3.83	3	**1.96**	0.63–6.08	2		exp 1.58	27	**1.77**	**1.21–2.57**
170	Breast	4	**1.61**	0.61–4.30	16	**1.13**	0.69–1.84	6	**0.53**	0.24–1.19	0		exp 1.94	74	**0.83**	0.67–1.05	100	**0.84**	0.69–1.03
171	Cervix uteri, women	0		exp 0.08	1		exp 0.76	0		exp 0.71	0		exp 0.10	5	**0.92**	0.38–2.21	6	**0.85**	0.38–1.88
172	Uterus, women	0		exp 0.10	0		exp 1.75	2		exp 1.40	1		exp 0.15	11	**0.74**	0.41–1.34	14	**0.77**	0.46–1.30
175	Ovaries women	0		exp 0.09	2		exp 1.26	2		exp 1.05	0		exp 0.13	11	**1.08**	0.60–1.96	15	**1.18**	0.71–1.96
177	Prostate, men	351	**1.08**	0.97–1.20	486	**1.18**	**1.08–1.29**	135	**0.67**	**0.57–0.80**	116	**0.98**	0.81–1.17	88	**0.84**	0.68–1.03	1176	**1.01**	0.96–1.07
178	Testis, men	5	**0.94**	0.39–2.26	4	**0.56**	0.21–1.48	9	**1.56**	0.81–2.99	1		exp 2.84	1		exp 1.74	20	**0.87**	0.56–1.36
180	Kidney	22	**0.69**	0.46–1.05	43	**1.05**	0.78–1.41	15	**0.74**	0.44–1.22	12	**1.00**	0.57–1.77	16	**1.04**	0.64–1.70	108	**0.90**	0.74–1.08
181	Urinary bladder	80	**1.12**	0.90–1.40	122	**1.31**	**1.10–1.57**	50	**1.18**	0.90–1.56	24	**0.93**	0.63–1.39	37	**1.27**	0.92–1.76	313	**1.20**	**1.07–1.34**
190	Melanoma	56	**1.06**	0.82–1.38	99	**1.44**	**1.18–1.75**	27	**0.72**	0.49–1.05	15	**0.71**	0.43–1.18	34	**1.11**	0.79–1.56	231	**1.10**	0.96–1.25
191	Other skin	85	**1.01**	0.81–1.24	184	**1.65**	**1.43–1.91**	27	**0.54**	**0.37–0.78**	22	**0.73**	0.48–1.11	38	**0.94**	0.69–1.29	356	**1.12**	**1.01–1.25**
193	Brain and nervous system	25	**1.00**	0.67–1.48	37	**1.12**	0.81–1.55	15	**0.80**	0.48–1.32	7	**0.68**	0.32–1.43	22	**1.32**	0.87–2.00	106	**1.02**	0.84–1.24
194	Thyroid	3		exp 4.40	6	**1.00**	0.45–2.22	6	**1.68**	0.75–3.73	0		exp 1.84	5	**1.11**	0.46–2.66	20	**0.98**	0.63–1.52
195	Other endocrine glands	9	**0.73**	0.38–1.40	17	**1.04**	0.64–1.67	6	**0.64**	0.29–1.43	5	**1.00**	0.41–2.39	10	**0.96**	0.52–1.79	47	**0.88**	0.66–1.17
197	Connective tissue	3		exp 5.94	8	**1.02**	0.51–2.03	8	**1.93**	0.96–3.85	1		exp 2.33	4	**1.20**	0.45–3.20	24	**1.02**	0.68‐1.52
199	Other and unspecified site	30	**1.36**	0.95–1.94	33	**1.11**	0.79–1.56	18	**1.31**	0.82–2.08	15	**1.86**	**1.12–3.08**	13	**0.89**	0.52–1.53	109	**1.24**	**1.02–1.49**
200, 202	Non‐Hodgkin's lymphoma	36	**0.97**	0.70–1.35	44	**0.91**	0.68–1.23	24	**1.00**	0.67–1.48	10	**0.71**	0.38–1.33	21	**1.09**	0.71–1.67	135	**0.95**	0.80–1.12
201	Hodgkin's disease	2		exp 3.01	3		exp 3.99	0		exp 2.48	1		exp 1.32	1		exp 1.60	7	**0.56**	0.27–1.18
203	Multiple myeloma	12	**0.84**	0.48–1.48	10	**0.54**	0.29–1.00	12	**1.34**	0.76–2.35	3		exp 5.24	5	**0.68**	0.28–1.63	42	**0.77**	0.57–1.04
204‐209	Leukemia	37	**1.00**	0.73–1.39	49	**1.01**	0.76–1.34	15	**0.63**	0.38–1.04	14	**1.01**	0.60–1.71	13	**0.66**	0.38–1.14	128	**0.90**	0.75–1.07

*Note:* Observed cases (O) after 5 years latency from start of sea service and 95% confidence intervals (CI) are given. Site analyzed if five cases or more for all categories, expected number of cases is shown if three cases or less (except for pleural mesothelioma).

*Never officers or engine crew.

Seafarers (genders combined) who started their sea service 1985 or later, working on different vessels, had an increased risk of thyroid cancer among the officers (SIR 2.58, 95% CI 1.23–5.41), stomach and lung cancer among the crew (SIR 2.57, 95% CI 1.16–5.72, SIR 1.92, 95% CI 1.16–3.18, respectively), and lung cancer among those with solely service (SIR 2.81, 95% CI 1.56–5.07; Table 4). The engine crew had a significantly increased risk of cancer of the mouth and pharynx (SIR 3.60; 95% CI 1.35–9.60), as well as lung cancer (SIR 2.74; 95% CI 1.23–6.10), unlike the deck crew (Supporting Information: Table III).

**Table 4:** Standardized incidence ratios (SIR) 1985–2020 by specific site for cancer (ICD‐7) and job category among Swedish seafarers (genders combined) with five years or more of registered signings, with start of sea service 1985–2013, and working on different types of vessels (not solely passenger ferries).

	Numbers (person‐years)	Officers *N* = 4203 (76,480 yrs)			Crew *N* = 3573 (61,253 yrs)				Solely service *N* = 1068 (22,008 yrs)			All categories *N* = 8844 (159,741 yrs)		
ICD‐7	**Cancer site** (if all ≥ 5 cases)	O	**SIR**	95% CI	O	**SIR**	95% CI		O	**SIR**	95% CI	O	**SIR**	95% CI
140‐209	All sites	225	**1.04**	0.91–1.18	167	**1.09**	0.94–1.27		86	**1.10**	0.89–1.36	478	**1.07**	0.97–1.16
141‐4	Mouth	4	**1.29**	0.48–3.43	5	**2.29**	0.95–5.50		0		exp 0.88	9	**1.46**	0.76–2.80
145‐8	Pharynx	2		exp 2.46	2		exp 1.69		1		exp 0.58	5	**1.06**	0.44–2.54
150	Esophagus	1		exp 2.14	2		exp 1.38		3		exp 0.41	6	**1.53**	0.69–3.40
151	Stomach	4	**1.15**	0.43–3.07	6	**2.57**	**1.16–5.72**		2		exp 0.88	12	**1.80**	**1.02–3.16**
153	Colon	13	**0.98**	0.57–1.69	9	**1.00**	0.52–1.92		5	**1.27**	0.53–3.06	27	**1.03**	0.71–1.50
154	Rectum	14	**1.59**	0.94–2.69	8	**1.34**	0.67–2.69		1		exp 2.47	23	**1.34**	0.89–2.01
155.0	Liver	4	**1.36**	0.51–3.62	2		exp 1.98		1		exp 0.65	7	**1.26**	0.60‐2.64
157	Pancreas	7	**1.83**	0.87–3.84	0		exp 2.58		1		exp 1.19	8	**1.05**	0.53–2.11
162.1	Lung	4	**0.34**	**0.13–0.92**	15	**1.92**	**1.16–3.18**		11	**2.81**	**1.56–5.07**	30	**1.28**	0.90–1.84
170	Breast	3		exp 3.24	3		exp 6.41		18	**0.84**	0.53–1.34	24	**0.77**	0.52–1.15
175	Ovaries, women	1		exp 0.19	0		exp 0.47		4	**2.39**	0.90–6.36	5	**2.14**	0.89–5.15
177	Prostate, men	51	**0.90**	0.68–1.18	31	**0.87**	0.61–1.24		6	**0.85**	0.38–1.89	88	**0.88**	0.72–1.09
178	Testis, men	9	**0.95**	0.50–1.83	10	**1.36**	0.73–2.53		1		exp 0.91	20	**1.13**	0.73–1.75
180	Kidney	11	**1.52**	0.84–2.75	8	**1.64**	0.82–3.27		2		exp 1.68	21	**1.52**	0.99–2.33
181	Urinary bladder	14	**1.23**	0.73‐2.08	7	**0.96**	0.46‐2.01		3		exp 2.07	24	**1.16**	0.78–1.73
190	Melanoma	23	**1.25**	0.83–1.89	14	**1.05**	0.62–1.77		6	**0.91**	0.41–2.03	43	**1.12**	0.83–1.51
191	Other skin	12	**0.90**	0.51–1.58	5	**0.58**	0.24–1.40		3		exp 3.34	20	**0.79**	0.51–1.23
193	Brain and nervous system	9	**0.95**	0.49–1.82	6	**0.84**	0.38–1.87		5	**1.51**	0.63–3.63	20	**1.00**	0.65–1.55
194	Thyroid	7	**2.58**	**1.23–5.41**	0		exp 2.29		2		exp 1.60	9	**1.36**	0.71–2.62
195	Other endocrine glands	6	**1.20**	0.54–2.68	4	**1.03**	0.39–2.74		2		exp 2.20	12	**1.08**	0.61–1.91
199	Other and unspecified site	5	**1.46**	0.61–3.51	4	**1.68**	0.63–4.47		1		exp 1.23	10	**1.42**	0.76–2.64
200, 202	Non‐Hodgkin's lymphoma	4	**0.46**	0.17–1.21	7	**1.15**	0.55–2.41		1		exp 2.28	12	**0.70**	0.40–1.23
201	Hodgkin's disease	4	**2.01**	0.76–5.36	1		exp 1.61		0		exp 0.44	5	**1.24**	0.51–2.97
204‐209	Leukemia	8	**0.91**	0.45–1.81	7	**1.14**	0.54–2.38		2		exp 2.39	17	**0.98**	0.61–1.57

*Note:* Observed cases (O) after 5 years latency from start of sea service and 95% confidence intervals (CI) are given. Site analyzed if 5 cases or more for all categories, expected number of cases is shown if three cases or less. Lip cancer for all categories: Four cases SIR 4.38 (95% CI 1.64–11.7).

### 3.4 Passenger Ferries

Those who had worked solely on passenger ferries did not have an increased total cancer risk (SIR 0.93; 95% CI 0.86–1.00; Table 5). However, seafarers who had worked solely with service on passenger ferries had an increased risk of mouth cancer (SIR 2.24; 95% CI 1.30–3.86) and lung cancer (1.43; 95% CI 1.05–1.96; Table 5).

**Table 5:** Standardized incidence ratios (SIR) 1985–2020 by specific site for cancer (ICD‐7), job category among Swedish seafarers (genders combined) with five years or more of registered signings, with start of sea service 1985–2013 and working solely on passenger ferries.

	Number (person‐years)	Officers *N* = 649 (11,147 yrs)			Crew *N* = 1589 (25,947 yrs)				Solely service *N* = 6932 (134,841 yrs)			All categories *N* = 9170 (171,934 yrs)		
ICD‐7	**Cancer site** (if all ≥ 5 cases)	O	**SIR**	95% CI	O	**SIR**	95% CI		O	**SIR**	95% CI	O	**SIR**	95% CI
140‐209	All sites	94	**1.02**	0.83–1.25	135	**1.02**	0.86–1.20		464	**0.89**	**0.81–0.97**	693	**0.93**	0.86–1.00
141‐144	Mouth	2		exp 1.11	1		exp 1.59		13	**2.24**	**1.30–3.86**	16	**1.88**	**1.15–3.07**
151	Stomach	1		exp 1.59	1		exp 2.06		4	**0.66**	0.25–1.77	6	**0.62**	0.28–1.38
153	Colon	4	**0.65**	0.24–1.72	4	**0.48**	0.18–1.27		22	**0.81**	0.54–1.24	30	**0.72**	0.50–1.03
154	Rectum	6	**1.53**	0.69–3.40	3		exp 5.21		15	**0.90**	0.54–1.50	24	**0.93**	0.63–1.39
157	Pancreas	3		exp 1.91	3		exp 2.50		9	**1.10**	0.57–2.11	15	**1.19**	0.72–1.97
162.1	Lung	5	**0.78**	0.32–1.87	7	**0.83**	0.40–1.75		39	**1.43**	**1.05–1.96**	51	**1.21**	0.92–1.60
170	Breast	1		exp 1.21	11	**0.93**	0.52–1.68		116	**0.86**	0.71–1.03	128	**0.86**	0.72–1.02
171	Cervix uteri, women	0		exp 0.10	2		exp 1.14		8	**0.67**	0.34–1.34	10	**0.76**	0.41–1.41
172	Uterus, women	0		exp 0.11	2		exp 1.17		3		exp 13.5	5	**0.34**	**0.14–0.81**
175	Ovaries, women	0		exp 0.08	5	**5.58**	**2.32–13.4**		10	**0.94**	0.50–1.74	15	**1.29**	0.78–2.14
177	Prostate, men	31	**1.04**	0.73–1.48	32	**0.98**	0.70–1.39		45	**0.90**	0.67–1.21	108	**0.96**	0.80–1.16
178	Testis, men	0		exp 0.75	2		exp 1.83		4	**0.72**	0.27–1.91	6	**0.74**	0.33–1.64
180	Kidney	4	**1.41**	0.53–3.77	4	**1.08**	0.41–2.89		9	**0.80**	0.41–1.53	17	**0.95**	0.59–1.53
181	Urinary bladder	9	**1.49**	0.78–2.87	5	**0.70**	0.29–1.69		9	**0.62**	0.32–1.19	23	**0.83**	0.55–1.25
190	Melanoma	4	**0.73**	0.27–1.94	7	**0.77**	0.37–1.61		52	**1.24**	0.95–1.63	63	**1.12**	0.87–1.43
191	Other skin	4	**0.50**	0.19–1.32	11	**1.14**	0.63–2.06		16	**0.68**	0.41–1.10	31	**0.75**	0.53–1.07
193	Brain and nervous system	0		exp 2.27	5	**1.21**	0.50–2.90		18	**0.86**	0.54–1.36	23	**0.84**	0.56–1.26
194	Thyroid	1		exp 0.53	1		exp 1.55		5	**0.50**	0.21–1.21	7	**0.58**	0.28–1.22
195	Other endocrine glands	2		exp 1.26	4	**1.55**	0.58–4.12		5	**0.36**	**0.15–0.86**	11	**0.62**	0.34–1.12
197	Connective tissue	1		exp 0.52	0		exp 0.82		7	**2.10**	1.00–4.41	8	**1.71**	0.86–3.42
199	Other and unspecified site	2		exp 1.62	3		exp 2.24		7	**0.82**	0.39–1.72	12	**0.97**	0.55–1.70
200, 202	Non‐Hodgkin's lymphoma	4	**1.23**	0.46–3.27	4	**0.89**	0.33–2.36		10	**0.66**	0.35–1.22	18	**0.78**	0.49–1.24
203	Multiple myeloma	2		exp 1.25	1		exp 1.61		3		exp 4.92	6	**0.77**	0.35–1.72
204‐209	Leukemia	2		exp 3.40	5	**1.06**	0.44–2.55		13	**0.81**	0.47–1.40	20	**0.83**	0.54–1.29

*Note:* Observed cases (O) after 5 years latency from start of sea service and 95% confidence intervals (CI) are given. Site analyzed if five cases or more for all categories, expected number of cases is shown if three cases or less. Pleura (mesothelioma) for all categories: four cases SIR 3.30 (95% CI 1.24–8.80).

## 4 Discussion

This study of cancer incidence in merchant seafarers in Sweden 1985–2020 confirms that Swedish seafarers had an increased overall cancer risk compared with the general population, particularly seafarers who worked on different vessels and started working before 1985. Since the risks in general were not significantly elevated for seafarers who started after 1985, this might imply a decreased risk of cancer in later years. This could be due to factors such as improved working conditions at sea, better access to health care, or lifestyle changes. However, since we lack reliable data on carcinogenic exposures among seafarers in more recent years, it is important to continue monitoring cancer incidence in this group.

Lifestyle factors for seafarers are also affected by the special conditions prevailing on the vessels, and that there may not be a sharp line between work and leisure time. For example, the diet is dependent on what the cook serves, and the degree of physical activity may be affected by what training opportunities are offered on the vessel. The availability of alcohol and tobacco on the vessels can also have an impact on seafarers' risk of cancer.

The risk for some cancer forms seems to decrease over time, for example lung and liver cancer in men. However, the risk for lung cancer in women, and lip, thyroid and stomach cancer in men, was still significantly increased in those who signed in 1985 or later, but the confidence intervals were wide as the risk estimates are based on a relatively small number of cases (12 cases of lung cancer in women, and four cases of lip, nine cases of thyroid, and 11 cases of stomach cancer in men).

Cancer incidence among the deck crew signing before 1985 was not increased (SIR 0.98, Table 3). It is possible that this could be a consequence of individuals succumbing to other diseases before many types of cancer have had time to develop, as cancer often has a very long latency period. A previous study on Swedish seafarers from the same cohort has shown an increased mortality in deck crew, both from all diseases (SMR 1.65, 95% CI 1.49–1.83) and external causes (SMR 2.54, 95% CI 2.14–3.03) [18].

Lung cancer incidence was increased in all job categories, probably mainly a result of a larger proportion of tobacco smokers among seafarers. Tobacco smoking increases the risk of a wide range of cancers, such as different types of cancers of the gastrointestinal tract, the urogenital system, and the respiratory airways, liver and pancreas cancer, and myeloid leukemia [19]. In recent years, the proportion of smokers has been about as low among Swedish seafarers as in the general population, but during the 1960s, about 65%–70% of Swedish seafarers were smokers, compared to around 50% in the general population [8, 9]. Research from a Swedish academic dissertation from 1998 found that 67% of the male deck officers, 51% of the male engineering officers, and 35% of the male engineering crew in Sweden smoked in 1977, compared to 44% of the male population at large [20]. A more recent study from 2017 showed that eleven percent of Swedish seafarers were smokers, and 33% were former smokers [9]. In those signing in 1985 or later in our study, lung cancer incidence was increased in those working with solely service, and in engine crew working on different vessels, but not in officers or deck crew (Supporting Information: Table III). It is not known whether smoking habits differ between deck crew and engine crew in our cohort. However, part of the difference in lung cancer incidence between officers, deck crew and engine crew could be due to exposure to occupational carcinogens in the engine room, such as asbestos, and some polycyclic aromatic hydrocarbons (PAHs) in soot and diesel exhausts, which are also well‐known causes of lung cancer in humans (IARC group 1). As was discussed in the previous study on this cohort, co‐exposure to tobacco smoke and occupational carcinogens might lead to effect interaction, that is, it could lead to a higher relative risk than if the separate effects were added together [7]. The phenomenon of effect variation in epidemiology is best known from studies of lung cancer risk in those who have been exposed to both asbestos and tobacco smoke [21]. Regarding other cancer forms associated with tobacco smoking, we also found an increased risk of cancer of the mouth, the pharynx, larynx, esophagus, stomach, liver, and urinary bladder, in men signing before 1985. The results are in accordance with population‐based cohort studies from Denmark 2005 and 2018, which showed that seafarers had an increased overall cancer risk, and particularly increased risk for lung cancer and other cancer forms associated with tobacco smoking [4].

Deck officers who started their sea service before 1985 had an increased risk of melanoma and other types of skin cancer in the present study; they may be more exposed to solar radiation, as this is a well‐documented risk factor for skin cancer [14]. The latency time from exposure to onset of disease can be very long for skin cancer [22]. A previous cohort study of cancer incidence among Finnish seafarers 1967–1992 also found an increased risk of non‐melanoma skin cancer, as well as an increased risk of mesothelioma, but no increased total cancer risk [1]. A later Finnish case‐control study showed that engine crew had an increased risk of mesothelioma and lung cancer [2].

The Finnish case–control study also indicated that deck crew on tankers had an increased risk of renal cancer, leukemia, and possibly lymphoma [2]. In a 2015 study from Germany, seafarers were more likely to have been hospitalized due to cancer, particularly leukemia, non‐Hodgkin lymphoma, respiratory cancer, and non‐melanoma skin‐cancer [6]. A study of hematologic malignancies in Swedish deck crew on tankers showed an increased incidence of lymphatic and hematopoietic cancer during 1961–1987, but a later study found no increased risk among those working on tankers from 1985 and later [8, 12]. In the present study, we found no increased risk of renal cancer, leukemia or lymphoma.

In a 2003 cohort‐study from Iceland, male marine engineers had an increased risk of mesothelioma and cancer of the lung, stomach, and urinary bladder [3]. In our study, engine officers (the most common position for marine engineers) had an increased risk of both mesothelioma and lung cancer, but not bladder cancer (Table 3).

One strength of the current study was that we only included seafarers in the study with at least five years of sea service. In this way, we automatically obtained a latency period of five years from the first exposure, which we believe is appropriate considering that cancers often do not appear until several years after the harmful exposure. In many cases, long‐term exposure is probably also required for the disease to occur. In addition, 42% (16,135) of the participants had worked for more than 20 years. We thereby reduced the risk of including those who only had short‐term and thereby perhaps not relevant exposure. In the previous study on this cohort, all seafarers with at least one sea service between 1985 and 2011, and with a total number of only ≥ 30 days of sea service, were included and followed until 2011 [7]. Another strength is that we used Swedish national registers with high coverage, reliability, and accuracy. One limitation with the study is that we do not have access to individual habits such as smoking, alcohol consumption and the amount of physical exercise. Another limitation is that the majority of the cohort consisted of men, as there are generally fewer women seafarers, resulting in limited statistical power to demonstrate a significantly increased or decreased cancer incidence in women. Despite this, we still have a relatively high proportion of women in our study compared to many other studies on seafarers.

There might also be a healthy worker effect, as all seafarers need a valid medical certificate that states that they are medically fit to perform their duties at sea.

## 5 Conclusion

Merchant seafarers in Sweden still have an increased overall cancer risk compared with the Swedish general population, particularly those who have been working on different types of vessels (not solely passenger ferries). There are many indications that the cancer risk among seafarers has decreased in recent years, probably due to improved conditions on board the vessels, including tighter restrictions on alcohol consumption on board and less tobacco smoking. However, this does not seem to apply to all types of cancer, and it is important to continue to follow their development and continuously work to improve the work environment and living conditions at sea.

## Author Contributions

Maria Wallin: Literature review, interpretation of the results, writing of the manuscript. Helena P. Eriksson: Previous studies on the cohort, analysis of the data, interpretation of the results, revision of the manuscript. Karl Forsell: Assistance in establishing the cohort, especially in verifying service details, revision of the manuscript. Eva Andersson: Establishment of the cohort, conception or design of the work, statistical calculations, interpretation of the results, revision of the manuscript. All authors discussed the results and contributed to and approved the final manuscript.

## Ethics Statement

The work was performed at the Department of Occupational and Environmental Medicine, Sahlgrenska Academy, University of Gothenburg, Gothenburg, Sweden. The Regional Ethic Committee of Gothenburg approved the study (193‐13) and the Swedish Ethical Review Authority approved the update of the study (2023‐06423‐02). Informed consent was not relevant as the data from the Seafarers' Register was linked to the Swedish Cancer Register by the National Board of Health and Welfare and then anonymized before we were given access to data.

## Conflicts of Interest

The authors declare no conflicts of interest.

## Supporting information


**Supporting table I:** Proportion of individuals with experience in a certain vessel type/job category who have also served on other vessel types/in other job categories, among Swedish seafarers with at least 5 years of sea service any time from 1985 to 2013 (but also before).
**Supporting table II:** Mean percentage of time worked in different job categories among those who started working as seafarers before 1985 and working on different types of vessels (i.e. those in Table III, representing most of the cancer cases). Proportions of the classified job categories that have most of their sea service time on passenger ferries are also shown.
**Supporting table III:** Standardized incidence ratios (SIR) 1985–2020 (genders combined) by specific sites of cancer (ICD‐7), job category, and year of first signing (before 1985 or 1985–2013) among Swedish seafarers with 5 years or more of registered signings and working on different types of vessels.

## Data Availability

The data that support the findings of this study are available on request from the corresponding author. The data are not publicly available due to privacy or ethical restrictions.
