# Supplementary material for: Cancer Incidence Among Swedish Seafarers Between 1985 and 2020
Source: Am J Ind Med. 2026 Aug 15;69(11):960–71. doi: 10.1002/ajim.70126 (PMC13649953; doi:10.1002/ajim.70126)
Supplement: Supplementary file 1 [file AJIM-69-960-s001.docx]

**Supplement table I.** Proportion of individuals with experience in a certain vessel type/job category who have also served on other vessel types/in other job categories, among Swedish seafarers with at least 5 years of sea service any time from 1985 to 2013 (but also before).

|  | **Only one type** | **Proportion of individuals with experience in a certain vessel type who also served on other vessel types, % of N** | | | | |  |
| --- | --- | --- | --- | --- | --- | --- | --- |
| **Vessel type*** |  | **Type A** | **Type F** | **Type O** | **Type P** | **Type T** | **N** |
| Ever type A | 8.7% |  | 8.9% | 74.9% | 78.0% | 97.8% | 5094 |
| Ever type F | 0.5% | 45.0% |  | 78.0% | 78.7% | 97.2% | 1004 |
| Ever type O | 4.2% | 26.0% | 5.5% |  | 77.4% | 86.1% | 14 707 |
| Ever type P | 39.5% | 12.6% | 2.5% | 36.0% |  | 56.0% | 31 613 |
| Ever type T | 9.7% | 21.6% | 4.2% | 54.8% | 76.6% |  | 23 101 |
| Total number |  |  |  |  |  |  | 38 030 |
|  | **Only one job cate-gory** | **Proportion of individuals with experience in a certain job category who also served in other categories, % of N** | | | | |  |
| **Job category** |  | **Deck officer** | **Deck crew** | **Engine officer** | **Engine crew** | **Service** | **N** |
| Ever deck officer | 9.9% |  | 84.5% | 6.4% | 8.8% | 20.4% | 9160 |
| Ever deck crew | 27.4% | 42.4% |  | 9.5% | 19.2% | 26.3% | 18 242 |
| Ever engine officer | 9.5% | 10.2% | 30.0% |  | 81.8% | 19.2% | 5786 |
| Ever engine crew | 13.8% | 9.3% | 40.8% | 55.1% |  | 24.2% | 8601 |
| Service | 67.4% | 9.8% | 25.1% | 5.8% | 10.9& |  | 19 100 |
| Total number |  |  |  |  |  |  | 38 030 |

*Type A – different kinds of tankers, type F – fishing vessels, type O – working vessels and other vessels, type P – passenger ferries, type T – transportation vessels.

**Supplement table II.** Mean percentage of time worked in different job categories among those who started working as seafarers before 1985 and working on different types of vessels (i.e. those in Table III, representing most of the cancer cases). Proportions of the classified job categories that have most of their sea service time on passenger ferries are also shown.

| Time worked in different job categories at sea,  mean % of total sea time* | **Ever engine officer**  **N=4019** | **Engine crew**  **N=1827** | **Deck officer**  **N=5383** | **Deck crew**  **N=3469** | **Solely service**  **N=2694** |
| --- | --- | --- | --- | --- | --- |
| Time as engine officer | 65.7% | 0% | 0% | 0% | 0% |
| Time as engine crew | 22.5% | 64.6% | 0.5% | 0% | 0% |
| Time as deck officer | 5.1% | 0% | 73.1% | 0% | 0% |
| Time as deck crew | 4.4% | 26.4% | 23.3% | 85.0% | 0% |
| Time as service | 1.1% | 7.9% | 1.9% | 14.8% | 99.0% |
|  |  |  |  |  |  |
| Seafarers with most sea service time on passenger ferries, column % | 22.2% | 24.7% | 26.0% | 25.0% | 52.5% |

*Not summing up to exactly 100%.

Supplement table III. Standardized incidence ratios (SIR) 1985-2020 (genders combined) by specific sites of cancer (ICD-7), job category, and year of first signing (before 1985 or 1985-2013) among Swedish seafarers with 5 years or more of registered signings and working on different types of vessels.

|  |  | | **Ever engine officer** | | | **Deck officer** | | | **Deck crew*** | | | | **Engine crew** | | | | | **Solely service** | | | | **All categories** | | | |
| --- | --- | --- | --- | --- | --- | --- | --- | --- | --- | --- | --- | --- | --- | --- | --- | --- | --- | --- | --- | --- | --- | --- | --- | --- | --- |
| ICD-7 | Cancer site | O | | **SIR** | 95% CI | O | **SIR** | 95% CI | | O | **SIR** | 95% CI | | O | **SIR** | 95% CI | O | | **SIR** | 95% CI | O | | **SIR** | 95% CI |  |
| **Start of sea service before 1985, different vessels** | | | | | |  |  |  | |  |  |  | |  |  |  |  | |  |  |  | |  |  |  |
| Numbers (p-yrs) | | N=4019 (115 528 yrs) | | | | N=5383 (153 955 yrs) | | | | N=3469 (100 208 yrs) | | | | N=1827 (52 066 yrs) | | | N=2694 (76 285 yrs) | | | | N=17 392 (498 042 yrs) | | | |  |
| 140-209 | All sites | 1180 | | **1.16** | **1.09-1.23** | 1682 | **1.25** | **1.19-1.31** | | 649 | **0.98** | 0.91-1.06 | | 427 | **1.13** | **1.03-1.24** | 660 | | **1.11** | **1.03-1.20** | 4598 | | **1.15** | **1.12-1.18** |  |
| 141-8 | Mouth-pharynx | 30 | | **1.40** | 0.98-2.00 | 47 | **1.71** | **1.29-2.28** | | 25 | **1.69** | **1.14-2.50** | | 14 | **1.66** | 0.98-2.81 | 16 | | **1.56** | 0.95-2.54 | 132 | | **1.60** | **1.35-1.90** |  |
| 162.1 | Lung | 120 | | **1.50** | **1.25-1.79** | 158 | **1.50** | **1.28-1.75** | | 76 | **1.58** | **1.26-1.98** | | 57 | **1.98** | **1.53-2.57** | 81 | | **1.80** | **1.45-2.24** | 492 | | **1.60** | **1.47-1.75** |  |
| 177 | Prostate, men | 351 | | **1.08** | 0.97-1.20 | 486 | **1.18** | **1.08-1.29** | | 135 | **0.67** | **0.57-0.80** | | 116 | **0.98** | 0.81-1.17 | 88 | | **0.84** | 0.68-1.03 | 1176 | | **1.01** | 0.96-1.07 |  |
| 181 | Urinary bladder | 80 | | **1.12** | 0.90-1.40 | 122 | **1.31** | **1.10-1.57** | | 50 | **1.18** | 0.90-1.56 | | 24 | **0.93** | 0.63-1.39 | 37 | | **1.27** | 0.92-1.76 | 313 | | **1.20** | **1.07-1.34** |  |
|  |  |  | |  |  |  |  |  | |  |  |  | |  |  |  |  | |  |  |  | |  |  |  |
| **Start of sea service 1985-2013, different vessels** | | | | | |  |  |  | |  |  |  | |  |  |  |  | |  |  |  | |  |  |  |
| Numbers (p-yrs) | | N=1463 (26 394 yrs) | | | | N=2740 (50 086 yrs) | | | | N=2470 (42 538 yrs) | | | | N=1103 (18 715 yrs) | | | N=1068 (22 008 yrs) | | | | N=8844 (159 741 yrs) | | | |  |
| 140-209 | All sites | 73 | | **1.00** | 0.79-1.26 | 152 | **1.05** | 0.90-1.24 | | 125 | **1.13** | 0.95-1.35 | | 42 | **0.98** | 0.73-1.33 | 86 | | **1.10** | 0.89-1.36 | 478 | | **1.07** | 0.97-1.16 |  |
| 141-8 | Mouth-pharynx | 2 | |  | exp 1.87 | 4 | **1.09** | 0.41-2.89 | | 3 |  | exp 2.77 | | 4 | **3.60** | **1.35-9.60** | 1 | |  | exp 1.46 | 14 | | **1.28** | 0.76-2.17 |  |
| 162.1 | Lung | 3 | |  | exp 3.79 | 1 |  | exp 7.83 | | 9 | **1.60** | 0.83-3.08 | | 6 | **2.74** | **1.23-6.10** | 11 | | **2.81** | **1.56-5.07** | 30 | | **1.28** | 0.90-1.84 |  |
| 177 | Prostate, men | 18 | | **0.96** | 0.60-1.52 | 33 | **0.86** | 0.61-1.22 | | 23 | **0.92** | 0.61-1.38 | | 8 | **0.76** | 0.38-1.52 | 6 | | **0.85** | 0.38-1.89 | 88 | | **0.88** | 0.72-1.09 |  |
| 181 | Urinary bladder | 4 | | **1.06** | 0.40-2.83 | 10 | **1.31** | 0.71-2.44 | | 6 | **1.16** | 0.52-2.59 | | 1 |  | exp 2.15 | 3 | |  | exp 2.07 | 24 | | **1.16** | 0.78-1.73 |  |
|  |  |  | |  |  |  |  |  | |  |  |  | |  |  |  |  | |  |  |  | |  |  |  |
| **Start of sea service 1985-2013, solely passenger ferries** | | | | | | | |  | |  |  |  | |  |  |  |  | |  |  |  | |  |  |  |
| Numbers (p-yrs) | | N=239 (4405 yrs) | | | | N=410 (6742 yrs) | | | | N=1309 (20 956 yrs) | | | | N=280 (4991 yrs) | | | N=6932 (134 841 yrs) | | | | N=9170 (171 934 yrs) | | | |  |
| 140-209 | All sites | 35 | | **1.14** | 0.82-1.58 | 59 | **0.96** | 0.75-1.24 | | 111 | **1.03** | 0.86-1.24 | | 24 | **0.94** | 0.63-1.41 | 464 | | **0.89** | **0.81-0.97** | 693 | | **0.93** | 0.86-1.00 |  |
| 141-8 | Mouth-pharynx | 0 | |  | exp 0.69 | 2 |  | exp 1.29 | | 1 |  | exp 2.14 | | 1 |  | exp 0.60 | 14 | | **1.45** | 0.86-2.45 | 18 | | **1.25** | 0.79-1.99 |  |
| 162.1 | Lung | 1 | |  | exp 2.15 | 4 | **0.93** | 0.35-2.49 | | 5 | **0.74** | 0.31-1.78 | | 2 |  | exp 1.63 | 39 | | **1.43** | **1.05-1.96** | 51 | | **1.21** | 0.92-1.60 |  |
| 177 | Prostate, men | 10 | | **1.01** | 0.54-1.88 | 21 | **1.06** | 0.69-1.62 | | 25 | **1.00** | 0.67-1.48 | | 7 | **0.94** | 0.45-1.97 | 45 | | **0.90** | 0.67-1.21 | 108 | | **0.96** | 0.80-1.16 |  |
| 181 | Urinary bladder | 4 | | **1.96** | 0.74-5.23 | 5 | **1.25** | 0.52-3.01 | | 3 | **0.54** | 5.59 | | 2 |  | exp 1.50 | 9 | | **0.62** | 0.32-1.19 | 23 | | **0.83** | 0.55-1.25 |  |
|  |  |  | |  |  |  |  |  | |  |  |  | |  |  |  |  | |  |  |  | |  |  |  |

Note: For seafarers working solely on passenger ferries only those with start of sea service 1985-2013 are shown. Observed cases (O) after 5 years latency from start of sea service and 95% confidence intervals (CI) are given. Expected number of cases is shown if 3 cases or less.*Never officers or engine crew
